# Association of the Clínica Universidad de Navarra-Body Adiposity Estimator With Type 2 Diabetes: A Retrospective Cohort Study

**DOI:** 10.3389/ijph.2023.1606063

**Published:** 2023-09-21

**Authors:** Shuoji Geng, Xuejiao Chen, Kaizhi Bai, Jiacheng Ding, Haojie Li, Songhe Shi

**Affiliations:** Department of Epidemiology and Health Statistics, College of Public Health, Zhengzhou University, Zhengzhou, China

**Keywords:** middle-aged and older adults, CUN-BAE, body mass index, waist circumference, type 2 diabetes mellitus

## Abstract

**Objectives:** Clínica Universidad de Navarra-Body Adiposity Estimator (CUN-BAE) is considered to be a more accurate indicator of body fat estimation. We aimed to investigate the association of CUN-BAE with the risk of type 2 diabetes mellitus (T2DM) and to compare the strength of the association between CUN-BAE, body mass index (BMI), waist circumference (WC), waist-to-height ratio (WHtR) and T2DM.

**Methods:** The data were obtained from the annual health checkup database of residents in Xinzheng, China. From January 2011 to December 2021, 80,555 subjects aged ≥45 years met the inclusion criteria. Cox proportional hazard regression models were used to estimate the hazard ratios (HRs) and 95% confidence intervals (CIs) for CUN-BAE, BMI, WC, and WHtR in T2DM.

**Results:** During a mean follow-up of 6.26 years, T2DM occurred in 12,967 subjects. The multivariable-adjusted HRs (95% CIs) of T2DM (highest vs. reference group) were 1.994 (1.811–2.196) for CUN-BAE, 1.751 (1.665–1.842) for WC, 1.715 (1.631–1.804) for WHtR, and 1.510 (1.436–1.588) for BMI, respectively. In addition, the risk of T2DM increased with baseline CUN-BAE (HR: 1.374; 95% CI: 1.328, 1.421), WC (HR: 1.236; 95% CI: 1.215, 1.256), WHtR (HR: 1.228; 95% CI: 1.208, 1.248), and BMI (HR: 1.175; 95% CI: 1.156, 1.195).

**Conclusion:** Compared to BMI, WC or WHtR, CUN-BAE may more adequately reflect the adverse effects of adiposity on the risk of T2DM.

## Introduction

As a major chronic noncommunicable disease, diabetes can lead to substantial morbidity, mortality, and healthcare expenditures and has been a huge public health burden worldwide [1]. According to the 2021 International Diabetes Federation Diabetes Atlas, an estimated 537 million adults worldwide have diabetes, and direct health expenditures due to diabetes are already close to one trillion USD. China, the world’s largest developing country, has the largest number of people with diabetes (140 million in 2021), and it is estimated that this number will reach 175 million by 2045 [2]. Type 2 diabetes mellitus (T2DM) is the main type of diabetes accounting for 90%–95% of diabetes and is a preventable and controllable disease [3]. However, a previous study showed that about approximately half of people with T2DM do not suspect they have the disease until they are diagnosed [4]. Therefore, it is very important to identify people at risk for T2DM early and to reduce the global diabetes epidemic through a valid and reproducible diagnostic indicator [5].

Among several modifiable risk factors for diabetes, obesity is considered a major risk factor [6, 7]. In the general population, the most widely used measures to define excess body fatness (BF) are body mass index (BMI), waist circumference (WC), and waist-to-height ratio (WHtR). Previous studies have shown that higher BMI, WC, and WHtR are associated with an increased risk of type 2 diabetes mellitus (T2DM) [8, 9]. However, these anthropometric indicators have been criticized because they do not consider important factors related to adiposity, especially age, sex, and race [10, 11]. Therefore, a new practical adiposity index, the Clínica Universidad de Navarra-Body Adiposity Estimator (CUN-BAE), was proposed and is considered to be a more accurate indicator of body fat estimation [12]. This method of estimating body fat percentage is based on BMI, sex, and age of Caucasian subjects and has the highest correlation with body fat percent as measured by air displacement volume tracing compared to other anthropometric methods [12]. Previous findings have shown that CUN-BAE is more strongly associated with T2DM and fat-related cardiovascular risk factors than BMI or WC [13, 14]. However, previous CUN-BAE assessment studies have been limited to white populations and need to be extended to other populations to determine its applicability.

To date, no studies have explored the applicability of CUN-BAE in the Chinese population. Therefore, the purpose of this study was to investigate the association of CUN-BAE with the risk of T2DM in the Chinese middle-aged and elderly population and to compare the strength of the association between CUN-BAE, BMI, WC and WHtR and T2DM.

## Methods

### Study Design and Participants

This was a retrospective population-based cohort study. The data were obtained from the annual health checkup database for residents of the Electronic Health Management Center in Xinzheng, Henan Province, China. Xinzheng’s annual health check-up program for residents is an important part of China’s basic public health service program. Since the main population of annual health checkups are middle-aged and elderly people, this study referred to the China Health and Retirement Longitudinal Survey and selected middle-aged and elderly people aged 45 years and above as the research subjects. From January 2011 to December 2021, 121,346 subjects were eligible for the study. We excluded subjects with the following conditions: (1) diabetes at baseline (*n* = 23,046); (2) age <45 years (*n* = 17,435); (3) missing information for drinking, smoking, exercise, height, weight, WC, resting heart rate (RHR), systolic blood pressure (SBP) or diastolic blood pressure (DBP) (*n* = 310). The data screening flow chart is presented in Supplementary Figure S1. This study was approved by the Ethics Committee of Zhengzhou University (Reference Number: ZZUIRB2019-019), and informed consent was obtained from all participants.

### Data Collection

Demographic and clinical information was collected from participants at each health screening. Demographic information included sex, age, marital status, smoking, drinking, and physical activity. Marital status was divided into couple and single, where single included unmarried, divorced, and widowed. Smoking was defined as never and current or previous. Drinking was defined as never, occasionally, and daily. Physical activity was divided into four categories: never, occasionally, more than once a week and daily. Clinical data included anthropometric measurements, laboratory investigations, and self-reported disease history. The study subjects wore light clothing and bare feet for height, weight, and waist measurements. Body height and weight were measured via a standard digital weighing scale and stadiometer, respectively. WC was measured using a calibrated tape measure while the subject was standing and during slight expiration. BMI was calculated as weight (kg)/height ^2^(m). WHtR was calculated as WC (m)/height (m). CUN-BAE = −44.988 + (0.503 × age) + (10.689 × sex) + (3.172 × BMI) - (0.026 × BMI^2^) +(0.181 × BMI × sex) - (0.02 × BMI × age)-(0.005 × BMI^2^ × sex) + (0.00021 × BMI^2^ × age), where male = 0 and female = 1 for sex and age in years [12]. Blood samples were collected after subjects had fasted for at least 8 h to measure blood lipids and blood glucose using an automated biochemical analyzer (DIRUICS380, Changchun, China). Resting heart rate (RHR), SBP and DBP were measured twice after subjects had rested for at least 5 min in a seated position using an automatic sphygmomanometer (Omron HEM-7125, Kyoto, Japan) [15], and the mean value was recorded as the final result.

### Definition of T2DM

According to the Chinese guidelines for T2DM, diabetes in this study was defined as: (1) self-reported doctor-diagnosed diabetes, (2) fasting plasma glucose ≥7.0 mmol/L, (3) current treatment with antidiabetic medication [16].

### Statistical Analysis

Continuous variables were expressed as means and standard deviations (SDs). Categorical variables were expressed as the numbers and frequencies. The chi-square test for categorical variables and the *t*-test for continuous variables were used to compare the differences between two groups defined by T2DM. Cox proportional hazard regression models were used to estimate the hazard ratios (HRs) and 95% confidence intervals (CIs) for CUN-BAE, BMI, WC, and WHtR in T2DM after confirming that the proportionality assumption was not violated. CUN-BAE, BMI, WC, and WHtR were evaluated in the following ways: (1) as quartiles and (2) as a continuous variable. Two Cox regression models were established: Model 1 with adjustment for sex, age, and marital status; and Model 2 adjusted for confounders, including sex, age, marital status, smoking, drinking, physical activity, SBP, DBP, and RHR. The dose‒response association and the potentially nonlinear relationship of continuous CUN-BAE, BMI, WC, and WHtR with T2DM were explored by restricted cubic spline models with four knots. In addition, stratified analysis was performed by subgroups of age and sex using a Cox regression model to test the consistency of these relationships.

To compare the magnitude of risk estimates, we also calculated relative risks for per-SD changes in CUN-BAE, BMI, WC, and WHtR among the total population and subgroups, compared the overall fitness of the models by the Akaike’s Information Criterion (AIC) [17], and assessed the predictive performance of the models using the Consistency Index (C-index) [18]. Sensitivity analysis was performed to assess the robustness of the results after the exclusion of participants with ≥3 years of follow-up. The statistical analyses were performed using SPSS V 21 and R V 4.0.3. *p* < 0.05 for a two-sided test was considered statistically significant.

## Results

### Characteristics of the Study Population

The baseline characteristics of the study subjects with and without diabetes are presented in Table 1. Overall, 80,555 subjects were studied, 52.3% of whom were women and 47.7% of whom were men. The mean (standard deviation, SD) follow-up was 6.26 (2.99) years. After 503,271 person-years of follow-up, T2DM occurred in 12,967 participants, and the overall incidence of T2DM was 257.7/10,000 person-years. Subjects who developed T2DM had higher levels of CUN-BAE, BMI, WC, and WHtR than those who did not (*p* < 0.01). The correlations between CUN-BAE, BMI, WC, and WHtR are shown in Supplementary Table S1.

**TABLE 1:** Baseline characteristics of the study population with and without diabetes (Xinzheng, China, 2011).

Characteristics	Total (*n* = 80,555)	Nondiabetes (*n* = 67,588)	Diabetes (*n* = 12,967)	*p*-value
Age (years)				<0.001
Middle-aged (45–59)	26,490 (32.90)	22,770 (33.70)	3,720 (28.70)	
Younger elderly (60–74)	48,224 (59.90)	39,761 (58.80)	8,463 (65.30)	
Older adults (≥75)	5,841 (7.30)	5,057 (7.50)	784 (6.00)	
Sex (%)				0.009
Men	38,396 (47.70)	32,078 (47.50)	6,318 (48.70)	
Women	42,159 (52.30)	35,510 (52.50)	6,649 (51.30)	
Marital status (%)				0.102
Couple	68,459 (85.00)	57,378 (84.90)	11,081 (85.50)	
Single	12,096 (15.00)	10,210 (15.10)	1,886 (14.50)	
Smoking (%)				<0.001
Never	68,403 (84.90)	57,679 (85.30)	10,724 (82.70)	
Current or previous	12,152 (15.10)	9,909 (14.70)	2,243 (17.30)	
Drinking (%)				<0.001
Never	74,575 (92.60)	62,731 (92.80)	11,844 (91.30)	
Occasionally	3,536 (4.40)	2,894 (4.30)	642 (5.00)	
Daily	2,444 (3.00)	1,963 (2.90)	481 (3.70)	
Physical activity (%)				<0.001
Never	61,327 (76.13)	51,836 (76.70)	9,491 (73.20)	
Occasionally	3,705 (4.60)	3,062 (4.50)	643 (5.00)	
More than once a week	4,140 (5.14)	3,463 (5.10)	677 (5.20)	
Daily	11,383 (14.13)	9,227 (13.70)	2,156 (16.60)	
SBP (mmHg)	132.01 ± 6.13	131.72 ± 5.98	133.49 ± 6.88	<0.001
DBP (mmHg)	80.29 ± 2.70	80.20 ± 2.65	80.77 ± 2.94	<0.001
RHR	73.78 ± 1.93	73.77 ± 1.93	73.84 ± 2.47	<0.001
CUN-BAE	31.88 ± 3.38	31.80 ± 2.38	32.29 ± 3.39	0.005
BMI (kg/m^2^)	24.47 ± 1.03	24.39 ± 1.03	24.87 ± 1.09	<0.001
WC, cm	83.37 ± 2.74	83.10 ± 2.60	84.80 ± 2.96	<0.001
WHtR	0.52 ± 0.02	0.52 ± 0.02	0.53 ± 0.02	<0.001

Abbreviations: CUN-BAE, Clínica Universidad de Navarra-Body Adiposity Estimator; BMI, body mass index; WC, waist circumference; WHtR, waist-to-height ratio; SBP, systolic blood pressure; DBP, diastolic blood pressure; RHR, resting heart rate.

### Risk of Diabetes by Baseline CUN-BAE, BMI, WC, and WHtR

Table 2 presents the HRs and 95% CIs for the association of T2DM with the four indicators (CUN-BAE, BMI, WC, and WHtR) at baseline in the total population. In this study, CUN-BAE, BMI, WC, and WHtR were all positively associated with T2DM risk in a dose‒response relationship (*p* < 0.001). In the total population, after adjusting for other covariates, including age, sex, marital status, drinking, smoking, physical activity, SBP, DBP, and RHR, the cumulative risk of diabetes increased with baseline CUN-BAE, BMI, WC, and WHtR quartile (HR (95% CI): 1.252 (1.190–1.317), 1.620 (1.480–1.773), and 1.994 (1.811–2.196) for CUN-BAE, 1.150 (1.092–1.210), 1.199 (1.139–263), and 1.510 (1.436–1.588) for BMI, 1.193 (1.131–1.258), 1.388 (1.317–1.463), and 1.751 (1.665–1.842) for WC, and 1.161 (1.098–1.228), 1.340 (1.273–1.411), and 1.715 (1.631–1.804) for WHtR, for quartiles 2, 3, and 4 versus quartile 1, respectively).

**TABLE 2:** Association of baseline anthropometric indicators with type 2 diabetes in the general population (Xinzheng, China, 2011–2021).

		Diabetes	Pearson-years of follow-up	Incidence rate, per 10,000 pearson-year	Model 1 HR (95%CI)	Model 2 HR (95%CI)
CUN-BAE	<25.25	2,986	127,479	234.2	Reference	Reference
	25.25–32.54	3,403	122,344	278.2	1.305 (1.241–1.372)	1.252 (1.190–1.317)
	32.54–37.83	3,036	128,425	236.4	1.750 (1.600–1.915)	1.620 (1.480–1.773)
	≥37.83	3,542	125,023	283.3	2.233 (2.030–2.457)	1.994 (1.811–2.196)
*p*-value					<0.001	<0.001
BMI	<22.46	3,827	132,587	288.6	Reference	Reference
	22.46–24.06	3,229	128,963	250.4	1.167 (1.109–1.229)	1.150 (1.092–1.210)
	24.06–26.30	3,145	125,059	251.5	1.243 (1.181–1.308)	1.199 (1.139–1.263)
	≥26.30	3,827	116,662	328.0	1.610 (1.533–1.691)	1.510 (1.436–1.588)
*p*-value					<0.001	<0.001
WC	<78	2,544	131,694	193.2	Reference	Reference
	78–83	2,975	128,732	231.1	1.211 (1.148–1.277)	1.193 (1.131–1.258)
	83–89	3,262	121,258	269.0	1.429 (1.356–1.506)	1.388 (1.317–1.463)
	≥89	4,186	121,587	344.3	1.862 (1.771–1.957)	1.751 (1.665–1.842)
*p*-value					<0.001	<0.001
WHtR	<0.48	2,443	124,322	196.5	Reference	Reference
	0.48–0.51	2,506	109,670	228.5	1.175 (1.112–1.243)	1.161 (1.098–1.228)
	0.51–0.55	3,553	135,405	262.4	1.377 (1.308–1.450)	1.340 (1.273–1.411)
	≥0.55	4,465	133,874	333.5	1.817 (1.729–1.910)	1.715 (1.631–1.804)
*p*-value					<0.001	<0.001

t Per 1,000 person-years.

Model 1: Adjusted age, sex, marital status. The CUN-BAE analyses is not adjusted for age and gender as they are included in CUN-BAE.

Model 2: Model 1 plus smoking, drinking, physical activity, SBP, DBP, and RHR.

Abbreviations: HR, hazard ratio; SBP, systolic blood pressure; DBP, diastolic blood pressure; RHR, resting heart rate; CUN-BAE, Clínica Universidad de Navarra-Body Adiposity Estimator; BMI, body mass index; WC, waist circumference; WHtR, waist-to-height ratio.

### Restricted Cubic Spline Curves for Four Indicators and Diabetes Risk

**FIGURE 1:**
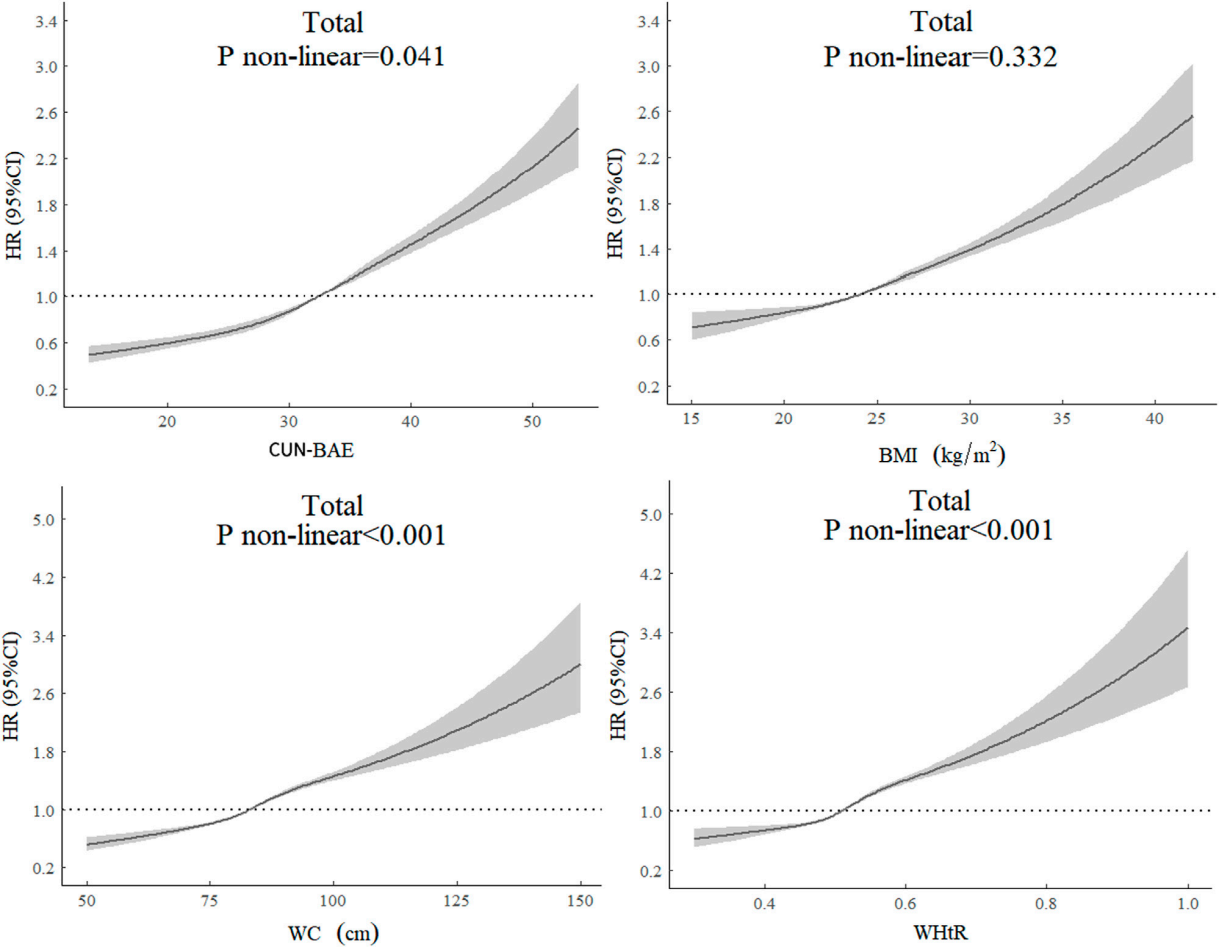
The dose‒response relationship between baseline anthropometric indicators and type 2 diabetes for all participants. HRs are adjusted for age, sex, smoking, drinking, physical exercise, SBP, DBP, and RHR. The CUN-BAE analyses is not adjusted for age and sex as they are included in CUN-BAE. Abbreviations: HR, hazard ratio; SBP, systolic blood pressure; DBP, diastolic blood pressure; RHR, resting heart rate; CUN-BAE, Clínica Universidad de Navarra-Body Adiposity Estimator; BMI, body mass index; WC, waist circumference; WHtR, waist-to-height ratio (Xinzheng, China, 2011–2021).

Multivariable adjusted restricted cubic spline analysis showed the dose‒response relationship between CUN-BAE, BMI, WC, WHtR, and T2DM for all participants and age stratification in Figure 1 and Supplementary Figures S2, S3, and the results showed that the risk of T2DM increased with increasing CUN-BAE, BMI, WC, and WHtR. The associations of CUN-BAE, WC, WHtR, and T2DM were nonlinear in all participants and in the young elderly group, but BMI and T2DM were approximately log-linear in all participants and age subgroups.

### Results of Subgroup Analyses and Sensitivity Analysis

**FIGURE 2:**
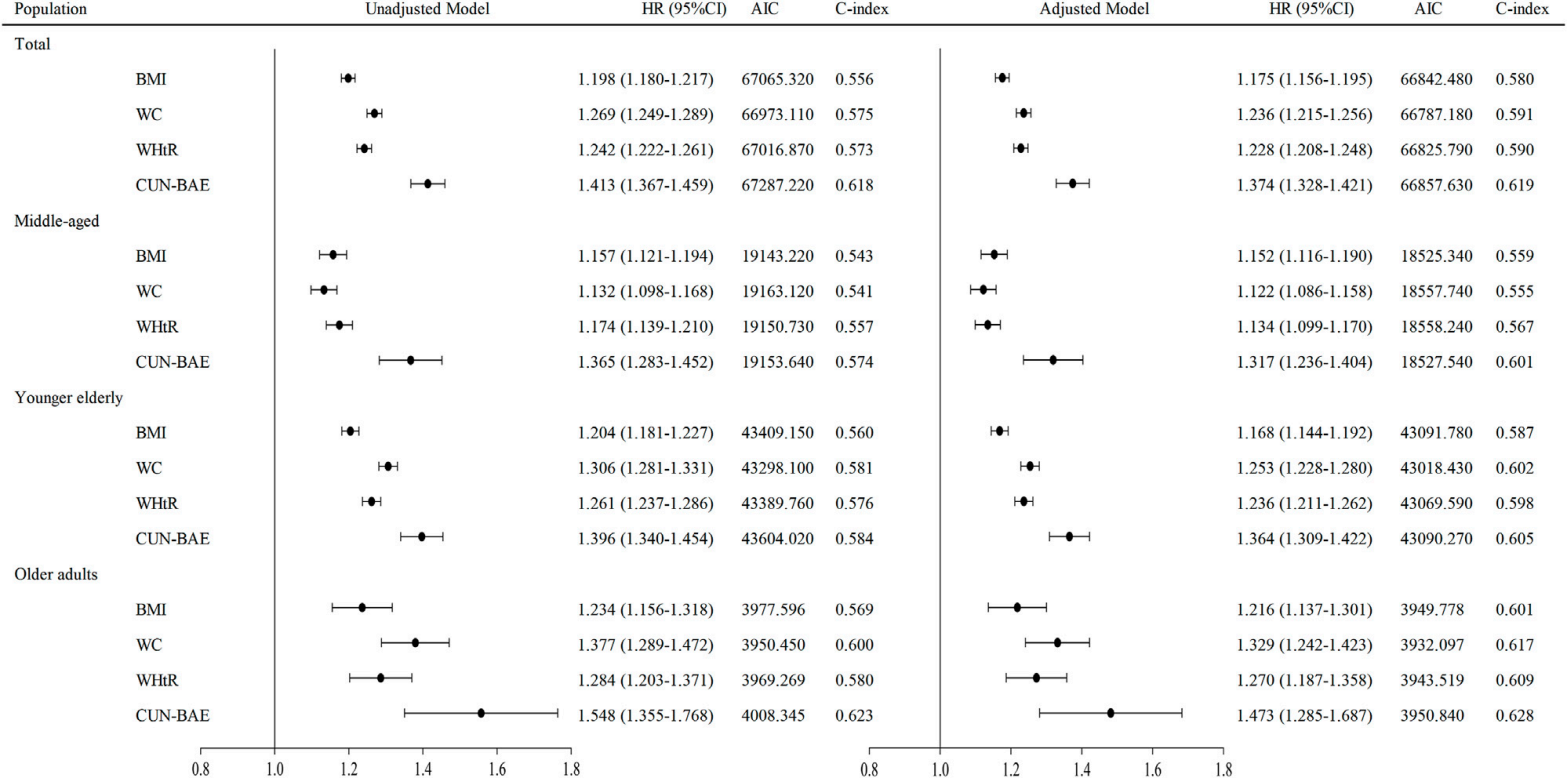
Risk of diabetes per SD increase in baseline anthropometric indicators. HRs are adjusted for age (not for age subgroup analysis), sex, smoking, drinking, physical exercise, SBP, DBP, and RHR. The CUN-BAE analyses is not adjusted for age and sex as they are included in CUN-BAE. Abbreviations: HR, hazard ratio; SBP, systolic blood pressure; DBP, diastolic blood pressure; RHR, resting heart rate; CUN-BAE, Clínica Universidad de Navarra-Body Adiposity Estimator; BMI, body mass index; WC, waist circumference; WHtR, waist-to-height ratio (Xinzheng, China, 2011–2021).

Stratified analysis by age subgroup also showed that higher CUN-BAE, BMI, WC, and WHtR were associated with a higher risk of T2DM in middle-aged, younger elderly and older adults (Tables 2 and 3). To compare the strength of the association of CUN-BAE, BMI, WC, or WHtR with diabetes, we calculated HRs (95% CIs) for T2DM with an increase of 1 standard deviation (SD) for each of the four indicators and assessed the overall fit of the model and the predictive power of the model using the AIC and the C-index, respectively (Figure 2). Among the total population, CUN-BAE was more significantly associated with T2DM risk than WC, WHtR, and BMI with multivariable-adjusted HRs (95% CIs) of T2DM being 1.374 (1.328–1.421) for CUN-BAE, 1.236 (1.215–1.256) for WC, 1.228 (1.208–1.248) for WHtR, and 1.175 (1.156–1.195) for BMI. In the age subgroup analysis, similar trends were observed in the younger elderly group aged 60–74 years and in the elderly group aged 75 years and older. Notably, a different trend was observed in the middle-aged group aged 45–59 years, where the association between BMI and T2DM was found to be weaker than CUN-BAE, but stronger than WC and WHtR. The results of the sex subgroup analysis are shown in Supplementary Table S2. The main results remained robust in sensitivity analyses after excluding participants with <3 years of follow-up (Supplementary Table S3).

**TABLE 3:** Association of baseline anthropometric indicators with type 2 diabetes in different age groups (Xinzheng, China, 2011–2021).

		Diabetes	Pearson-years of follow-up	Incidence rate, per 10,000 pearson-year	Model 1 HR (95% CI)	Model 2 HR (95% CI)
Middle-aged						
CUN-BAE	<24.67	951	43,659	217.8	Reference	Reference
	24.67–32.59	846	32,526	260.1	1.291 (1.173–1.421)	1.253 (1.138–1.379)
	32.59–37.09	1,038	46,732	222.1	1.527 (1.290–1.808)	1.448 (1.222–1.715)
	≥37.09	885	31,036	285.2	1.917 (1.612–2.279)	1.765 (1.482–2.103)
*p*-value					<0.001	<0.001
BMI	<22.84	881	30,659	287.4	Reference	Reference
	22.84–24.22	839	44,370	189.1	1.077 (0.980–1.184)	1.066 (0.970–1.172)
	24.22–26.37	952	42,584	223.6	1.154 (1.053–1.265)	1.123 (1.025–1.232)
	≥26.37	1,048	36,340	288.4	1.476 (1.349–1.615)	1.408 (1.285–1.542)
*p*-value					<0.001	<0.001
WC	<78	782	36,115	216.5	Reference	Reference
	78–83	957	42,014	227.8	1.101 (1.001–1.210)	1.084 (0.986–1.193)
	83–89	978	39,842	245.5	1.210 (1.100–1.330)	1.183 (1.075–1.301)
	≥89	1,003	25,982	386.0	1.393 (1.267–1.533)	1.330 (1.208–1.465)
*p*-value					<0.001	<0.001
WHtR	<0.48	847	40,131	211.1	Reference	Reference
	0.48–0.51	833	37,540	221.9	1.096 (0.996–1.206)	1.088 (0.988–1.197)
	0.51–0.54	1,052	43,274	243.1	1.182 (1.074–1.302)	1.160 (1.054–1.277)
	≥0.54	988	33,008	299.3	1.435 (1.314–1.568)	1.381 (1.262–1.510)
*p*-value					<0.001	<0.001
Younger elderly						
CUN-BAE	<25.24	1,950	78,796	247.5	Reference	Reference
	25.24–32.00	2,331	79,564	293.0	1.288 (1.211–1.369)	1.229 (1.155–1.308)
	32.00–37.97	1,840	71,909	255.9	1.899 (1.708–2.112)	1.730 (1.554–1.925)
	≥37.97	2,342	78,590	298.0	2.409 (2.144–2.706)	2.100 (1.866–2.364)
*p*-value					<0.001	<0.001
BMI	<22.31	1,738	86,089	201.9	Reference	Reference
	22.31–24.03	2,032	74,980	271.0	1.208 (1.133–1.288)	1.187 (1.113–1.266)
	24.03–26.34	2,158	74,603	289.3	1.277 (1.198–1.360)	1.228 (1.152–1.309)
	≥26.34	2,535	73,187	346.4	1.620 (1.524–1.723)	1.502 (1.411–1.599)
*p*-value					<0.001	<0.001
WC	<78	1,562	80,323	194.5	Reference	Reference
	78–83	1,829	76,778	238.2	1.210 (1.131–1.295)	1.188 (1.110–1.271)
	83–89	2,124	73,768	287.9	1.460 (1.367–1.559)	1.407 (1.317–1.503)
	≥89	2,948	77,990	378.0	1.954 (1.836–2.079)	1.815 (1.704–1.933)
*p*-value					<0.001	<0.001
WHtR	<0.48	1,447	73,072	198.0	Reference	Reference
	0.48–0.52	1,525	63,926	238.6	1.246 (1.166–1.332)	1.224 (1.145–1.308)
	0.52–0.56	2,304	82,377	279.7	1.441 (1.347–1.540)	1.381 (1.291–1.477)
	≥0.56	3,187	89,483	356.2	1.968 (1.845–2.099)	1.829 (1.713–1.952)
*p*-value					<0.001	<0.001
Older adults						
CUN-BAE	<27.79	85	5,024	169.2	Reference	Reference
	27.79–35.88	226	10,253	220.4	1.367 (1.098–1.702)	1.275 (1.022–1.591)
	35.88–39.55	158	9,785	161.5	1.287 (0.911–1.818)	1.200 (0.848–1.698)
	≥39.55	315	15,397	204.6	2.019 (1.441–2.827)	1.826 (1.298–2.568)
*p*-value					<0.001	<0.001
BMI	<21.37	160	15,839	101.0	Reference	Reference
	21.37–23.23	166	9,614	172.7	1.063 (0.855–1.321)	1.056 (0.849–1.313)
	23.23–25.43	206	7,872	261.7	1.319 (1.072–1.622)	1.282 (1.041–1.578)
	≥25.43	252	7,134	353.2	1.741 (1.427–2.123)	1.653 (1.351–2.022)
*p*-value					<0.001	<0.001
WC	<75	200	15,257	131.1	Reference	Reference
	75–80	189	9,940	190.1	1.401 (1.102–1.781)	1.387 (1.090–1.764)
	80–87	160	7,647	209.2	1.550 (1.247–1.926)	1.507 (1.211–1.875)
	≥87	235	7,615	308.6	2.329 (1.881–2.884)	2.154 (1.737–2.672)
*p*-value					<0.001	<0.001
WHtR	<0.47	149	11,119	134.0	Reference	Reference
	0.47–0.51	148	8,204	180.4	1.250 (0.991–1.577)	1.211 (0.958–1.529)
	0.51–0.56	197	9,755	201.9	1.563 (1.252–1.950)	1.487 (1.190–1.858)
	≥0.56	290	11,381	254.8	1.968 (1.580–2.451)	1.809 (1.450–2.257)
*p*-value					<0.001	<0.001

t Per 1,000 person-years.

Model 1: Adjusted sex, marital status. The CUN-BAE analyses is not adjusted for sex as it is included in CUN-BAE.

Model 2: Model 1 plus smoking, drinking, physical activity, SBP, DBP, and RHR.

Abbreviations: HR, hazard ratio; SBP, systolic blood pressure; DBP, diastolic blood pressure; RHR, resting heart rate; CUN-BAE, Clínica Universidad de Navarra-Body Adiposity Estimator; BMI, body mass index; WC, waist circumference; WHtR, waist-to-height ratio.

## Discussion

In this retrospective cohort study, we investigated the association between CUN-BAE and the risk of T2DM in a Chinese middle-aged and elderly population and compared the strength of the association between CUN-BAE and BMI, WC, and WHtR with T2DM. We found that increased CUN-BAE was associated with an increased risk of T2DM and that CUN-BAE was more strongly associated with the risk of T2DM than BMI, WC, or WHtR. The same results were found in the age subgroup analysis. We found that the association between BMI and T2DM was weaker than CUN-BAE but stronger than WC and WHtR in the middle-aged population, whereas the association between BMI and T2DM was found to be weaker than WC and WHtR in the young elderly and elderly individuals. Dose‒response relationships by restricted cubic spline analysis revealed a nonlinear relationship between CUN-BAE, WC, WHtR, and T2DM in the total population and in the younger elderly.

To our knowledge, this is the first large population-based cohort study to examine the relationship between CUN-BAE, a new practical adiposity index, and the risk of T2DM in the Chinese population. Due to the infeasibility of using expensive techniques to measure body composition at a large-scale population level, prior research has relied heavily on the use of anthropometric methods to examine the relationship between obesity and T2DM [19–21]. Traditionally, BMI has been the most commonly used measure of overall obesity, as its calculation requires only simple height and weight information. In addition, WC and WHtR, which are used to measure abdominal obesity, have also increasingly attracted the interest of researchers [22–24]. In a meta-analysis of 21 prospective studies and a meta-analysis of 216 cohort studies, BMI, WC, and WHtR showed a strong positive association with the risk of T2DM [9, 25]. Consistent with these findings, a strong positive association of BMI, WC, and WHtR with T2DM was also found in our study analysis. Several mechanisms can be used to explain this positive association. First, individuals with a genetic susceptibility to T2DM are more likely to be obese because the inherent insulin resistance in the muscle and islet α-cells of these individuals leads to increased glucose and insulin release in the liver, which results in obesity [26]. Second, chronic inflammation associated with obesity and its pro-inflammatory cytokines produced by macrophages in adipose tissue can affect insulin-dependent tissues and beta cells [27]. Third, a series of secretory products released from the stress adipocytes of obese individuals can lead to a loss of insulin sensitivity and beta-cell capacity of the pancreas [28]. Fourth, obese people tend to consume high-calorie foods and high-fat diets, which have been shown to lead to hypothalamic mitochondrial dysfunction and endoplasmic reticulum contingencies, thereby promoting leptin and insulin resistance, which results in T2DM [29]. In addition, BMI and WC or WHtR are considered to be fairly good measures of overall obesity and abdominal obesity, respectively, yet neither can distinguish well between fat mass and lean body mass. A recent study suggested that it is crucial to understand the independent roles of fat mass and lean body mass in mortality [30]. In this study, we were able to distinguish more precisely between fat mass and lean body mass using validated CUN-BAE metrics. We found that CUN-BAE was more associated with the risk of T2DM than BMI, WC and WHtR in middle-aged and older populations. This finding suggests that BMI, WC or WHtR do not fully reflect the extent to which fat mass is detrimental to the risk of type T2DM [31] and that CUN-BAE metrics may address this limitation.

BMI, WC, and WHtR represent various aspects of body composition, yet the consensus on which metric is most closely associated with diabetes risk is unclear. A meta-analysis that included 31 studies found that WHtR was more strongly associated with T2DM than BMI or WC, while another meta-analysis that included 21 cohort studies showed no significant difference in identifying diabetes risk between BMI, WC, and WHtR [25, 32]. In recent years, there has been increasing evidence that abdominal fat, rather than overall fat, might be a more associated risk factor for the development of diabetes [33, 34]. Our study found a stronger association of abdominal fat metrics, specifically WC, with the risk of T2DM compared to BMI. However, it is noteworthy that subgroup analysis stratified by age showed that a strong association of abdominal fat indicators was only observed in the older age group of 60 years and older, whereas WC and WHtR were found to have weaker associations with T2DM risk than BMI in the middle-aged population aged 45–59 years. This suggests that the effectiveness of overall obesity indicators and abdominal obesity indicators in screening people at risk for T2DM may vary across age groups. Previous studies have found that body composition changes with age, including increased fat mass, decreased muscle mass, redistribution of adipose tissue, and shrinkage in height [35], which may be used to explain our findings. In addition, our findings indicate that both WC and WHtR are strongly associated with the risk of developing T2DM, but WHtR was not materially superior to WC in the older age group of 60 years and older, suggesting that there is no additional benefit to measuring height other than WC in the older population.

When we compared CUN-BAE with BMI, WC, and WHtR, CUN-BAE showed the strongest association with T2DM risk, consistent with the results of studies in European populations [12, 13, 36]. In a cross-sectional study of Spanish adults aged 18–96 years, Veronica [36] found that the CUN-BAE index was more strongly associated with cardiometabolic conditions, including diabetes, arterial hypertension and metabolic syndrome (Mets), than BMI and WC, suggesting that CUN-BAE may better identify people at risk for cardiometabolic disease than BMI. Vicente [13] found that CUN-BAE was more strongly associated with diabetes than BMI in adult men. In addition, [37] showed that CUN-BAE had the strongest association with metabolic syndrome compared to BMI, WHtR and other indicators, suggesting that CUN-BAE can be used as an alternative to BMI for the initial screening of people at high risk of metabolic syndrome. Multivariable adjusted restricted cubic spline analysis showed that CUN-BAE, WC, WHtR, and T2DM were nonlinearly associated, but BMI and T2DM were approximately log-linear. The reason for this result may be their different ways of assessing body fat and fat distribution, with CUN-BAE, WC, and WHtR reflecting body fat distribution, whereas BMI mainly reflects overall body fat mass. In the subgroup analysis stratified by age, we found a stronger association between CUN-BAE, BMI, WC, WHtR, and T2DM in the older group than in the middle-aged group, suggesting that different indicators of obesity may perform better in different age groups. However, in the subgroup analysis stratified by sex, we found that the association between CUN-BAE and T2DM was attenuated and approached that of BMI and T2DM. A sex-stratified analysis of 9,555 Iranian subjects indicated similar modest associations between BMI and CUN-BAE with the risk of developing cardiovascular disease risk factors, including metabolic syndrome, hypercholesterolemia, and hypertension [38]. Paradoxically, a prospective cohort study of 6,796 individuals in Norway showed that CUN-BAE was more associated with the risk of CVD events and diabetes than BMI at the time of sex-stratified analysis [14]. The differences in our findings may reflect differences in study design (cross-sectional study vs. cohort study). This may also be related to differences in body composition between Asian and European populations. Overall, our study supports for the first time a strong correlation between the CUN-BAE index and the incidence of T2DM in the Chinese population. More studies are needed to further support our findings.

Our study has several strengths. First, the large sample size, the long follow-up period, the standardized measures used, and the use of an annual health examination dataset in this study avoided recall bias to some extent. Second, we compared the differences in the association between obesity indicators and T2DM risk in different subgroups by age and sex subgroup analysis. Finally, a sensitivity analysis was performed to assess the robustness of the association between obesity indicators and the risk of developing T2DM. However, there are some limitations of this study that should be noted. First, this study focused on the middle-aged and elderly population, and it was therefore not possible to compare the relationship between obesity indicators and T2DM risk in other age groups, which limited the generalizability of this study. Second, although many confounding factors were adjusted for in the analysis of this study, there were still some potential confounding factors present that were not adjusted for, such as literacy and dietary habits. Finally, the dose‒response correlations should be considered with caution because our study sample limited the generalizability of our results.

### Conclusion

Our study found that increased CUN-BAE was associated with an increased risk of T2DM in the Chinese middle-aged and elderly population and that CUN-BAE was more strongly associated with T2DM risk than BMI, WC, or WHtR. The same results were found in the analysis of age stratification. Our findings suggest that CUN-BAE may more adequately reflect the adverse effects of adiposity on T2DM risk than BMI, WC, or WHtR.
