# Supplementary material for: Association of the Clínica Universidad de Navarra-Body Adiposity Estimator With Type 2 Diabetes: A Retrospective Cohort Study
Source: Int J Public Health. 2023 Sep 21;68:1606063. doi: 10.3389/ijph.2023.1606063 (PMC10551821; doi:10.3389/ijph.2023.1606063)
Supplement: Supplementary file 1 [file DataSheet1.docx]

**
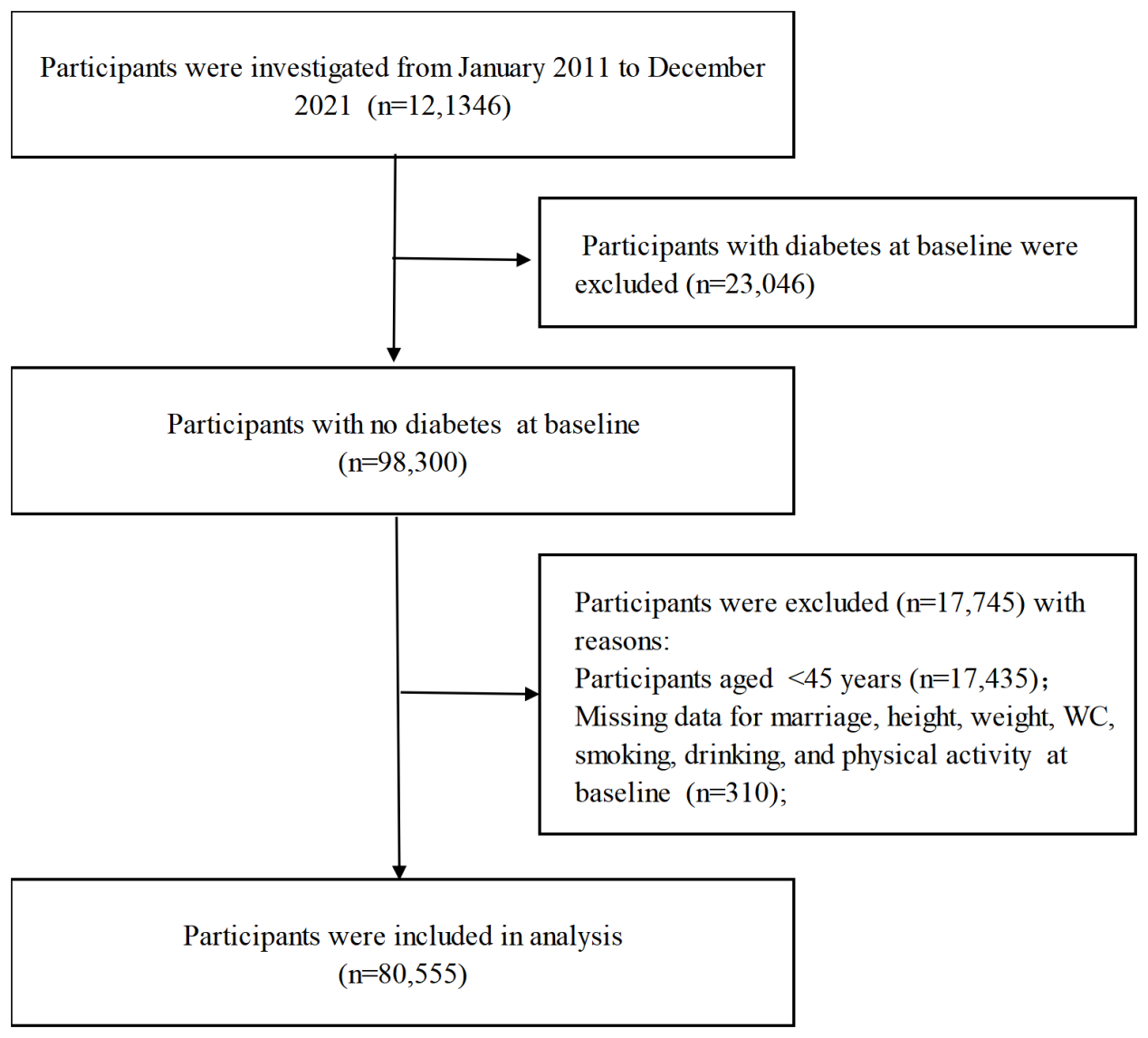
**

**SUPPLEMENTARY FIGURE 1** Flow diagram of participant selection (Xinzheng, China, 2011).

**
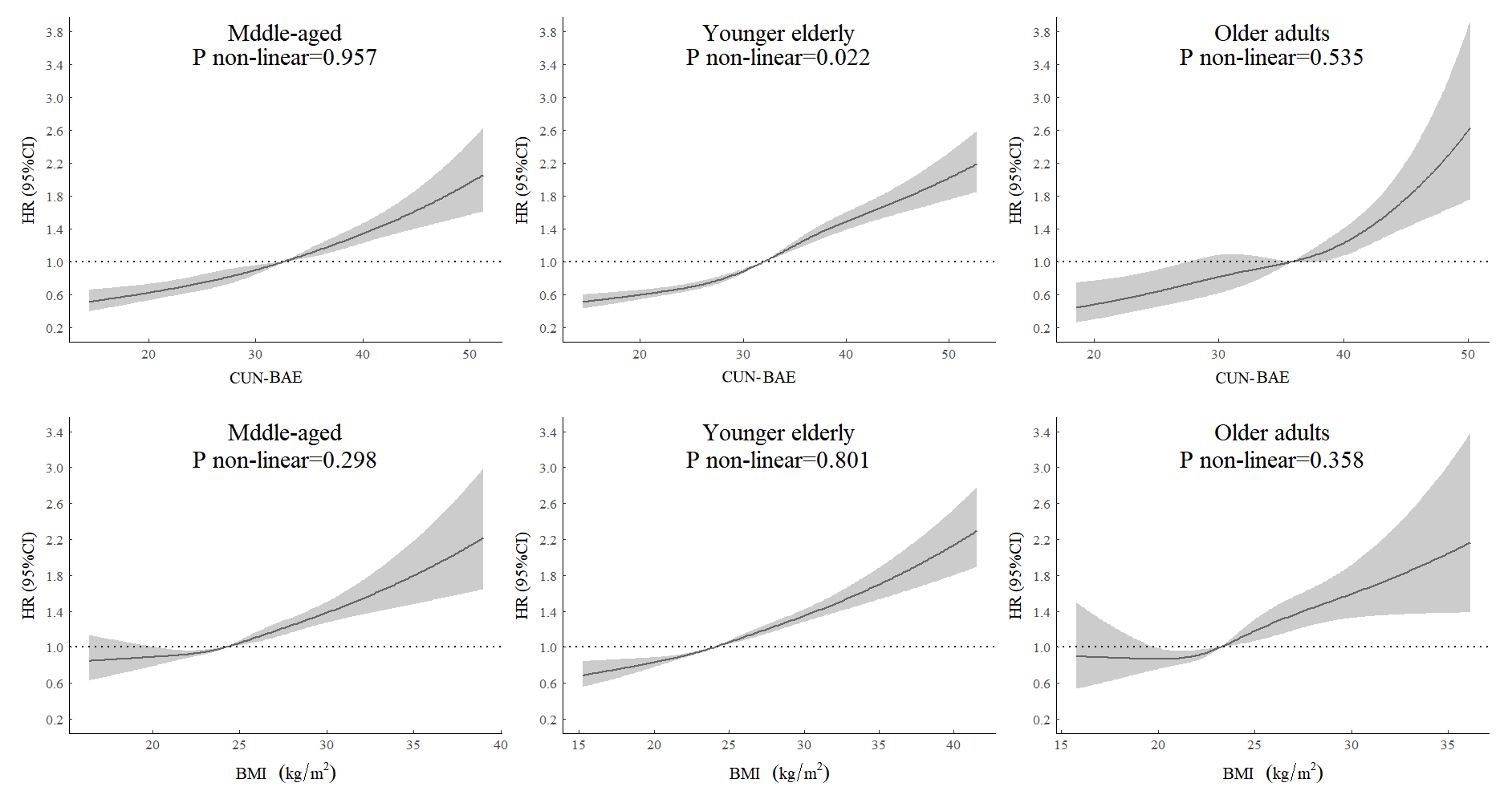
SUPPLEMENTARY FIGURE 2** Association of type 2 diabetes and Clínica Universidad de Navarra-Body Adiposity Estimator and body mass index by age groups. Adjusted Model: HRs are adjusted for sex, Marital status, drinking, smoking, physical activity, SBP, DBP, and RHR. The CUN-BAE analyses is not adjusted for sex as it is included in CUN-BAE. Abbreviations: HR, hazard ratio; SBP, systolic blood pressure; DBP, diastolic blood pressure; RHR, resting heart rate; CUN-BAE, Clínica Universidad de Navarra-Body Adiposity Estimator; BMI, body mass index; WC, waist circumference; WHtR, waist-to-height ratio (Xinzheng, China, 2011-2021).

**SUPPLEMENTARY FIGURE
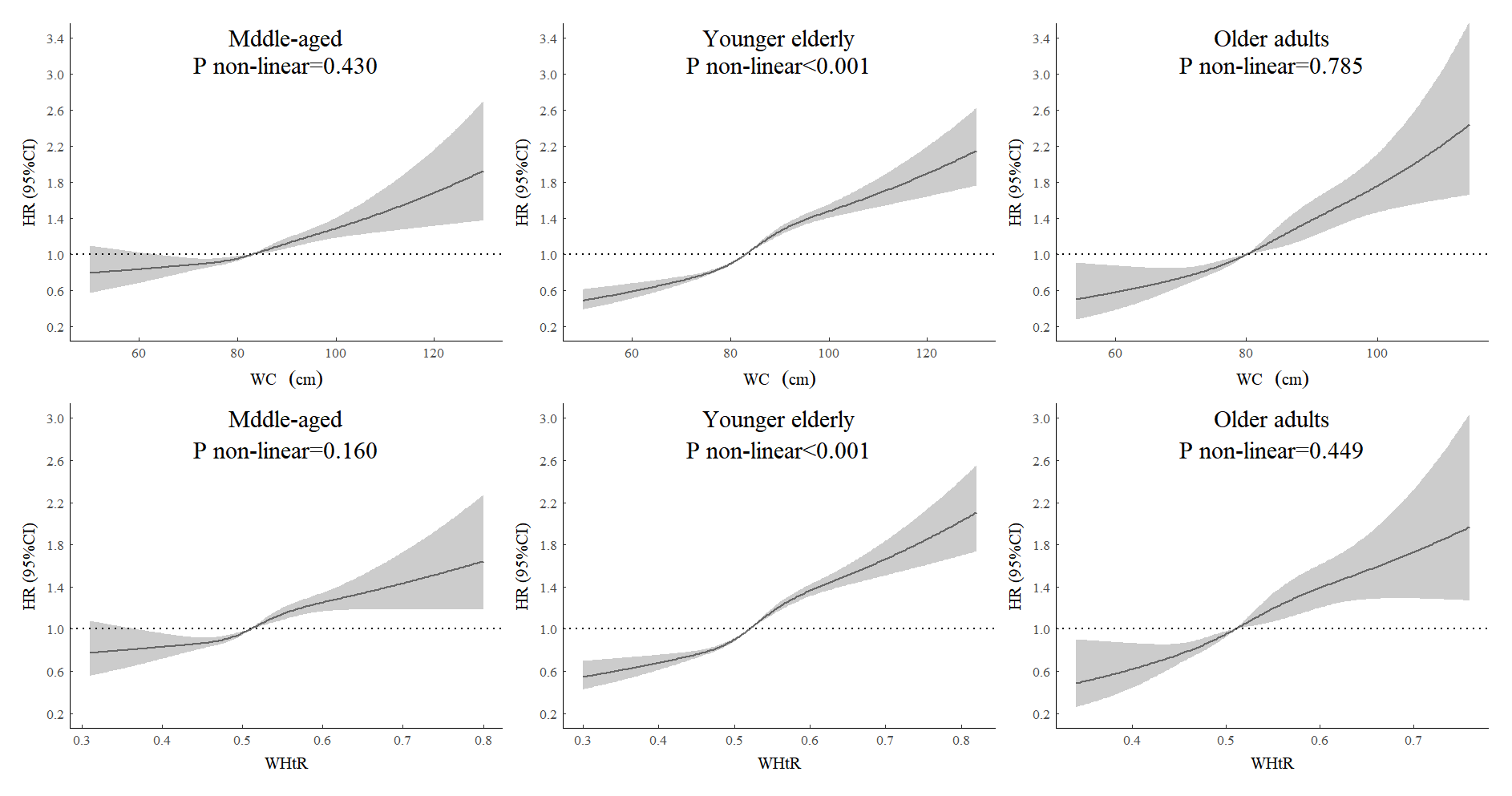
 3** Association of type 2 diabetes and waist circumference and waist-to-height ratio by age groups. Adjusted Model: HRs are adjusted for gender, Marital status, drinking, smoking, physical activity, SBP, DBP, and RHR. Abbreviations: HR, hazard ratio; SBP, systolic blood pressure; DBP, diastolic blood pressure; RHR, resting heart rate; CUN-BAE, Clínica Universidad de Navarra-Body Adiposity Estimator; BMI, body mass index; WC, waist circumference; WHtR, waist-to-height ratio (Xinzheng, China, 2011-2021).

| **SUPPLEMENTARY TABLE 1 The Pearson correlations of all anthropometric indices (Xinzheng, China, 2011).** | | | | |
| --- | --- | --- | --- | --- |
| Indices | CUN-BAE | BMI | WC | WHtR |
| CUN-BAE | 1 | 0.547* | 0.134* | 0.397* |
| BMI | 0.547* | 1 | 0.526* | 0.558* |
| WC | 0.134* | 0.526* | 1 | 0.896* |
| WHtR | 0.397* | 0.558* | 0.896* | 1 |

Abbreviations: CUN-BAE, Clínica Universidad de Navarra-Body Adiposity Estimator; BMI, body mass index; WC, waist circumference; WHtR, waist-to-height ratio.

*:*p* < 0.001, statistically significant.

| **SUPPLEMENTARY TABLE 2 Association of baseline anthropometric indicators with type 2 diabetes risk after stratification by sex (Xinzheng, China, 2011-2021).** | | | | | | | | | | | | | |
| --- | --- | --- | --- | --- | --- | --- | --- | --- | --- | --- | --- | --- | --- |
|  | **Male** | **Diabetes** | **Pearson-years** **of** **follow-up** | **Incidence rate,** **per** **10,000** **pearson-year** | **Model** **1 HR**  **(95%CI)** | **Model** **2 HR**  **(95%CI)** |  | **Female** | **Diabetes** | **Pearson-years** **of** **follow-up** | **Incidence** **rate,** **per** **10,000** **pearson-year** | **Model** **1 HR**  **(95%CI)** | **Model** **2 HR**  **(95%CI)** |
| CUN-BAE | < 22.78 | 1,351 | 60,621 | 222.9 | Reference | Reference | CUN-BAE | < 35.21 | 1,315 | 67,869 | 193.8 | Reference | Reference |
|  | 22.78–25.03 | 1,490 | 61,031 | 244.1 | 1.103 (1.024– 1.187) | 1.082 (1.005– 1.165) |  | 35.21–37.58 | 1,651 | 67,759 | 243.7 | 1.283 (1.193– 1.380) | 1.268 (1.179– 1.364) |
|  | 25.03–27.72 | 1,586 | 59,643 | 265.9 | 1.216 (1.130– 1.309) | 1.171 (1.087– 1.260) |  | 37.58–40.35 | 1,698 | 67,104 | 253.0 | 1.355 (1.260– 1.458) | 1.319 (1.226– 1.419) |
|  | ≥ 27.72 | 1,891 | 55,051 | 343.5 | 1.605 (1.495– 1.723) | 1.481 (1.378– 1.592) |  | ≥ 40.35 | 1,985 | 64,193 | 309.2 | 1.676 (1.561– 1.799) | 1.592 (1.481– 1.711) |
| *p*-value |  |  |  |  | <0.001 | <0.001 | *p*-value |  |  |  |  | <0.001 | <0.001 |
| Continuous (per SD) | |  |  |  | 1.211 (1.181– 1.241) | 1.175 (1.145– 1.205) | Continuous (per SD) | |  |  |  | 1.211 (1.182– 1.241) | 1.187 (1.158– 1.216) |
| BMI | <22.39 | 1,395 | 62,611 | 222.8 | Reference | Reference | BMI | <22.49 | 1,366 | 69,888 | 195.5 | Reference | Reference |
|  | 22.39-23.88 | 1,507 | 60,878 | 247.5 | 1.115 (1.037– 1.200) | 1.098 (1.021– 1.182) |  | 22.49-24.22 | 1,630 | 68,918 | 236.5 | 1.208 (1.124– 1.299) | 1.191 (1.107– 1.280) |
|  | 23.88-26.00 | 1,531 | 58,907 | 259.9 | 1.177 (1.094– 1.266) | 1.133 (1.053– 1.220) |  | 24.22-26.56 | 1,643 | 64,042 | 256.6 | 1.318 (1.226– 1.416) | 1.276 (1.187– 1.372) |
|  | ≥26.00 | 1,885 | 53,950 | 349.4 | 1.616 (1.507– 1.732) | 1.490 (1.388– 1.600) |  | ≥26.56 | 2,010 | 64,077 | 313.7 | 1.619 (1.511– 1.735) | 1.538 (1.434– 1.650) |
| *p*-value |  |  |  |  | <0.001 | <0.001 | *p*-value |  |  |  |  | <0.001 | <0.001 |
| Continuous (per SD) | |  |  |  | 1.202 (1.175– 1.229) | 1.172 (1.144– 1.200) | Continuous (per SD) | |  |  |  | 1.200 (1.173– 1.227) | 1.178 (1.152– 1.206) |
| WC | < 80 | 1,319 | 61,855 | 213.2 | Reference | Reference | WC | < 76 | 1,268 | 71,576 | 177.2 | Reference | Reference |
|  | 80–85 | 1,437 | 59,979 | 239.6 | 1.143 (1.060– 1.232) | 1.118 (1.037– 1.205) |  | 76–81 | 1,519 | 68,049 | 223.2 | 1.276 (1.184– 1.375) | 1.263 (1.172– 1.361) |
|  | 85–90 | 1,464 | 53670 | 272.8 | 1.320 (1.225– 1.422) | 1.268 (1.176– 1.367) |  | 81–88 | 1,778 | 66,324 | 268.1 | 1.550 (1.442– 1.667) | 1.518 (1.412– 1.632) |
|  | ≥ 90 | 2,098 | 60,842 | 344.8 | 1.699 (1.585– 1.821) | 1.569 (1.462– 1.683) |  | ≥ 88 | 2,084 | 60,976 | 341.8 | 2.010 (1.874– 2.156) | 1.919 (1.788– 2.060) |
| *p*-value |  |  |  |  | <0.001 | <0.001 | *p*-value |  |  |  |  | <0.001 | <0.001 |
| Continuous (per SD) | |  |  |  | 1.241 (1.213– 1.269) | 1.207 (1.179– 1.236) | Continuous (per SD) | |  |  |  | 1.282 (1.253– 1.311) | 1.256 (1.228– 1.286) |
| WHtR | < 0.48 | 1,386 | 64,478 | 215.0 | Reference | Reference | WHtR | < 0.48 | 1,057 | 59,844 | 176.6 | Reference | Reference |
|  | 0.48–0.51 | 1,371 | 57,387 | 236.9 | 1.126 (1.045– 1.213) | 1.108 (1.028– 1.194) |  | 0.48–0.52 | 1,579 | 70,770 | 223.1 | 1.279 (1.183– 1.382) | 1.267 (1.172– 1.370) |
|  | 0.51–0.54 | 1,382 | 52,185 | 264.8 | 1.269 (1.177– 1.367) | 1.230 (1.141– 1.325) |  | 0.52–0.56 | 1,667 | 65,145 | 255.9 | 1.486 (1.376– 1.605) | 1.455 (1.347– 1.572) |
|  | ≥ 0.54 | 2,179 | 62,296 | 349.8 | 1.713 (1.602– 1.833) | 1.586 (1.480– 1.698) |  | ≥ 0.56 | 2,346 | 71,166 | 329.7 | 1.963 (1.825– 2.111) | 1.879 (1.746– 2.022) |
| *p*-value |  |  |  |  | <0.001 | <0.001 | *p*-value |  |  |  |  | <0.001 | <0.001 |
| Continuous (per SD) | |  |  |  | 1.236 (1.209– 1.264) | 1.204 (1.176– 1.232) | Continuous (per SD) | |  |  |  | 1.269 (1.241– 1.298) | 1.245 (1.217– 1.274) |
| t Per 1,000 person-years.  Model 1: Adjusted age, marital status. The CUN-BAE analyses is not adjusted for age as it is included in CUN-BAE.  Model 2: Model 1 plus smoking, alcohol consumption, physical activity, SBP, DBP, and RHR.  Abbreviations: HR, hazard ratio;SBP, systolic blood pressure; DBP, diastolic blood pressure; RHR, resting heart rate; CUN-BAE, Clínica Universidad de Navarra-Body Adiposity Estimator; BMI, body mass index; WC, waist circumference; WHtR, waist-to-height ratio. | | | | | | | | | | | | | |

| **SUPPLEMENTARY TABLE 3 Association between baseline anthropometric indicators and diabetes in study subjects with ≥3 years of follow-up (Xinzheng, China, 2011-2021).** | | | | | | |
| --- | --- | --- | --- | --- | --- | --- |
|  |  | **Diabetes** | **Pearson-years** **of** **follow-up** | **Incidence rate,** **per** **10,000** **pearson-year** | **Model** **1 HR**  **(95%CI)** | **Model** **2 HR**  **(95%CI)** |
| CUN-BAE | < 25.16 | 2,156 | 118,814 | 181.5 | Reference | Reference |
|  | 25.16–32.62 | 2,414 | 117,948 | 204.7 | 1.225 (1.154– 1.301) | 1.194 (1.125– 1.268) |
|  | 32.62–37.79 | 2,094 | 119,726 | 174.9 | 1.491 (1.338– 1.662) | 1.419 (1.273– 1.582) |
|  | ≥ 37.79 | 2,431 | 118,670 | 204.9 | 1.845 (1.647– 2.066) | 1.719 (1.533– 1.928) |
| *p*-value |  |  |  |  | <0.001 | <0.001 |
| Continuous (per SD) | |  |  |  | 1.354 (1.300– 1.409) | 1.316 (1.264– 1.371) |
| BMI | <22.32 | 1,912 | 121,568 | 157.3 | Reference | Reference |
|  | 22.32-23.92 | 2,227 | 119,449 | 186.4 | 1.189 (1.118– 1.264) | 1.178 (1.107– 1.252) |
|  | 23.92-26.12 | 2,268 | 118,164 | 191.9 | 1.231 (1.158– 1.309) | 1.207 (1.135– 1.284) |
|  | ≥26.12 | 2,688 | 115,977 | 231.8 | 1.528 (1.440– 1.621) | 1.161 (1.138– 1.184) |
| *p*-value |  |  |  |  | <0.001 | <0.001 |
| Continuous (per SD) | |  |  |  | 1.176 (1.153– 1.199) | 1.161 (1.138– 1.184) |
| WC | < 77 | 1,774 | 118,959 | 149.1 | Reference | Reference |
|  | 77–82 | 1,953 | 110,353 | 177.0 | 1.211 (1.135– 1.292) | 1.199 (1.124– 1.279) |
|  | 82–89 | 2,605 | 133,726 | 194.8 | 1.365 (1.284– 1.451) | 1.338 (1.259– 1.422) |
|  | ≥ 89 | 2,763 | 112,120 | 246.4 | 1.794 (1.689– 1.906) | 1.718 (1.616– 1.826) |
| *p*-value |  |  |  |  | <0.001 | <0.001 |
| Continuous (per SD) | |  |  |  | 1.242 (1.218– 1.266) | 1.222 (1.198– 1.247) |
| WHtR | < 0.48 | 1,881 | 119,810 | 157.0 | Reference | Reference |
|  | 0.48–0.51 | 1,857 | 104,486 | 177.7 | 1.158 (1.086– 1.235) | 1.147 (1.076– 1.223) |
|  | 0.51–0.55 | 2,491 | 127,427 | 195.5 | 1.314 (1.237– 1.395) | 1.288 (1.213– 1.368) |
|  | ≥ 0.55 | 2,866 | 123,435 | 232.2 | 1.647 (1.553– 1.746) | 1.579 (1.488– 1.675) |
| *p*-value |  |  |  |  | <0.001 | <0.001 |
| Continuous (per SD) | |  |  |  | 1.219 (1.196– 1.243) | 1.200 (1.177– 1.225) |
| t Per 1,000 person-years.  Model 1: Adjusted age, gender, marital status.  Model 2: Model 1 plus smoking, drinking, physical activity, SBP, DBP, and RHR.  Abbreviations: HR, hazard ratio; SBP, systolic blood pressure; DBP, diastolic blood pressure; RHR, resting heart rate; CUN-BAE, Clínica Universidad de Navarra-Body Adiposity Estimator; BMI, body mass index; WC, waist circumference; WHtR, waist-to-height ratio. | | | | | | |
